# Drive and Instinct—How They Produce Relatedness and Addiction

**DOI:** 10.3389/fpsyg.2021.657944

**Published:** 2021-06-10

**Authors:** Thomas Ringwood, Lindsay Cox, Breanna Felldin, Michael Kirsch, Brian Johnson

**Affiliations:** ^1^Department of Psychiatry, State University of New York (SUNY) Upstate Medical University, Syracuse, NY, United States; ^2^Institute of Physiological Chemistry, University Hospital Essen, Essen, Germany

**Keywords:** psychoanalaysis, neuropsychoanalysis, addiction, drive, instinct, mass psychology

## Abstract

Addictive drugs are responsible for mass killing. Neither persons with addiction nor the general populace seem conscious of the malevolence of governments and drug dealers working together. How could this be? What is the place of psychoanalysis in thinking about deaths from addiction and in responding to patients with addiction? To answer these questions, we revise concepts of SEEKING, drive, instinct, pleasure, and unpleasure as separable. We review the neurobiological mechanism of cathexis. We discuss how addictive drugs take over the will by changing the SEEKING system. We review how opioid tone in the central nervous system regulates human relationships and how this endogenous hormonal system is modified by external opioid administration. We differentiate the pleasure of relatedness from the unpleasure of urgent need including the urgent need for drugs. We show how addictive drug-induced changes in the SEEKING system diminish dopaminergic tone, reducing the motivation to engage in the pursuit of food, water, sex, sleep, and relationships in favor of addictive drugs. With this neuropsychoanalytic understanding of how drugs work, we become more confidently conscious of our ability to respond individually and socially.

## Introduction

Twenty-three percent of Americans die from drug addiction, 17% tobacco, 3% alcohol, 2% opioids, and 1% other drugs (CDC websites)^1^^,^ ^2^^,^ ^3^^,^ ^4^ (Table 1). The first two are sold with government approval and participation through licensing and taxation. Although illicit drug dealers are responsible for overdose deaths, medical providers also supply opioid and benzodiazepine medications that are also responsible for many overdose deaths.

**Table 1:** Deaths in the United States from drugs−2018.

**Cause of death**		**Percentage**
Overall	2,839,205	100
Tobacco	480,000	16.9
Alcohol	95,000	3.3
Drug overdose	67,367	2.4
Total addiction deaths	642,367	22.6

Most people are not aware of the killing. Worldwide tobacco killed 100 million in the 20th century and is on track to kill 1 billion in the 21st (Koh, 2016). At the very least, one would imagine that psychoanalysis might address how mass killing occurs constantly and appears to be outside the conscious awareness of most humans.

Does psychoanalysis have anything to say about addiction treatment or is it a biological disorder that is outside the purview of a clinical pursuit that depends on interpersonal discussions? Should a psychoanalyst who realizes that their patient has chemical dependency refer the patient out to a drug counselor and an addiction treatment program?

The heart of psychoanalysis involves examining the relationship between the treater and the patient. Difficulties observed there can be addressed to solve barriers to relatedness in general. Perhaps, we might start there.

## Human Relationships as a Solution to Addictive Disorders

Addiction presents a unique challenge in that it is both common in the population and yet remains, despite advances in understanding its underlying genetic, neurobiological, and social risk factors, stubbornly difficult to treat. Does this complicated phenomenon warrant complicated treatment? Maybe not. Alcoholics Anonymous (AA), as described by its members, is “A simple program for complicated people.” A 2020 Cochrane Review concludes that interventions aimed at increasing AA engagement and participation (12-Step Facilitation) consistently lead to higher rates of continuous abstinence, indicating that long-term abstinence is not necessarily derived from Twelve-Step Facilitation interventions, but from continued participation in the AA fellowship (Kelly et al., 2020). Although AA has a spiritual tone, many members do not hold the spiritual tenants of the program as essential for recovery (Alcoholics Anonymous, 2018; Kelly et al., 2018). Instead, they see the 12-step program as simultaneously offering involvement in a social fellowship and facilitating a character change necessary to develop and sustain fulfilling human relationships (Alcoholics Anonymous, 2001). There are 12-step programs for drugs, tobacco, gambling, and over-eating, as well as other fellowship-based organizations, such as Self-Management and Recovery Training (SMART) and Women for Sobriety for people with addictions; these programs may prove to have the same benefits demonstrated in AA (Kelly et al., 2020). In other words, AA and related fellowships offer a simple solution, human connection, to the complicated phenomenon of addiction.

What can be said about how the human connection facilitated by 12-step programs, such as AA, serves as an effective treatment for addiction? Neuropsychoanalytic thinking provides insight into addiction and recovery as being related to human connection. Addiction can be seen as an attempt to substitute a drug experience/drug for persons (Johnson, 1999, 2003). Previous publications have created a theoretical hierarchy based on neuropsychoanalytic premises that depression is the biologically determined response to social isolation (Watt and Panksepp, 2009). The concept that addiction exists to ward off depression and conflicts with dependency has been extensively developed by Mosri (2019). Humans are social animals with an innate drive to be attached and related to others (Johnson, 2008). We fall ill when we become disconnected and unrelated. Other attempts at not depending on others are narcissism and psychopathy, fantasies of relationships with an ideal self and other that either follow social norms, narcissism, or make rules that are invented by the person, psychopathy (Johnson, 1993). In the transference focused psychotherapy (Kernberg, 2016) practiced on our addiction medicine service, difficulties with relatedness routinely enter the transference relationship when drug use stops (Johnson, 1992).

Our effort to apply the answers to relatedness and addiction uses the neuropsychoanalytic premise that neuroscience is the basic science of psychoanalysis (Johnson and Mosri, 2016). We take psychoanalysis as a general psychology, as the scientific study of human subjectivity (Koh, 2016).

We will begin with a review of drive as described by Michael Kirsch (Kirsch and Mertens, 2018; Kirsch, 2019). Kirsch explained that all drives depend on hormones that lodge in the lateral hypothalamus to switch the SEEKING system from hunger to thirst to sex to sleep. We will then describe hormonal systems involving endogenous opioids and explain sequential engagement of dopaminergic drive, oxytocin, and opioid stimulation as a foundation of cathexis/relatedness with classical switching in the lateral hypothalamus. These neurobiological systems are disrupted by drugs. With this theoretical understanding, we will understand more fully why AA is so effective, and why psychoanalytic examination of relatedness is often of help in recovery from addiction.

## Neuropsychoanalytic Understanding of Relatedness and of Addiction

### Drive: Hormones as the Imperative Motor Factor

How can an unconscious motivation induce a conscious emotion or promote a behavior? Kirsch (2019) discussed this concept as one that is relatively accepted within scientific communities but states that the mechanism by which specific motivations may induce specific emotions is not fully understood. He identified Freud's motivational drives as an example of these “unconscious motivations” and asked *how* such motivations might lead to conscious emotions. In Freud's literature on his theory of motivational drives, he identified hunger, thirst, and sex drive as three drives that are similar in that they all have a unique somatic stimulus that acts to promote a specific behavior (Kirsch, 2019). He called these stimuli “imperative motor factors.” In 1902, the first hormone was discovered, and in 1905, Freud posited that his imperative motor factors were likely to be “chemical messengers” or hormones (Kirsch, 2019). Kirsch was intrigued by Freud's identification of motivational drives and sought to determine the neurobiological similarities between these so-called “Freudian” drives in order to determine both whether he could identify a neurobiological avenue by which unconscious motivations can lead to conscious emotions, as well as whether there were other Freudian drives (2019).

Kirsch described three criteria of a Freudian motivational drive—they have an imperative nature, they originate in the lateral hypothalamus, and 5-hydroxytryptamine acts to downregulate the drive and is released once the drive has been satisfied (2019). Using these criteria, Kirsch identified a fourth motivational drive, sleep (2019). He correlated each drive with a unique hormone/imperative motor factor that enabled him to demonstrate that motivational drives are produced independently of one another and target drive-specific areas of the brain (Kirsch, 2019). He correlated hunger with ghrelin, thirst with angiotensin II, sex drive with testosterone/estradiol, and sleep with adenosine. In addition to each hormone targeting brain areas specific to its corresponding drive, Kirsch demonstrated that each hormone targets the lateral hypothalamus and the nucleus accumbens, two main brain areas associated with Panksepp's dopaminergic SEEKING system (2019). Both areas are acted on directly by all four Freudian imperative motor factors—ghrelin, testosterone, angiotensin II, and adenosine. This process requires that peripherally released hormones are able to cross the blood–brain barrier in order to act upon specific brain areas and, in turn, induce the release of neurotransmitters and neuromodulators (Kirsch, 2019). The release of neurotransmitters and neuromodulators is thought to be the way in which peripheral messages can be “converted” into conscious ones (Kirsch, 2019). It is thus theorized that this is the mechanism by which unconscious Freudian motivational drives are able to induce conscious emotions.

Johnson (2008) described the mechanism of cathexis, the psychoanalytic word for persons that means something to one another. Reviewing the animal literature on bonding, Johnson explained that the first dopaminergic SEEKING must be engaged, and then oxytocin and endogenous opioid must be expressed at the same time. If one of these three, dopamine, oxytocin, or endogenous opioid, is blocked, there is no cathexis. 5-Hydroxytryptamine is responsible for processing attachment (Kirsch and Buchholz, 2020).

Reviewing more neurobiology, Johnson (2013) found that “drive reduction” requires sequential engagement of dopaminergic SEEKING followed by gratification. He used Freud's concept of “the will” as drives acting within us. We want to seek and find gratification of drives. It is what all animals want. Finally, drugs take over the will by becoming a goal of the SEEKING system. Drugs are wanted more than natural (food, water, sex, relationships, sleep) goals of the will.

### SEEKING, Drive, and Instinct

Panksepp conceptualized the SEEKING system as a dopaminergic pathway that modulates the need both for specific instincts (hunger, thirst, and sex) that originate in the lateral hypothalamus, as well as his other emotional systems, RAGE, FEAR, PANIC, CARE, LUST, and PLAY (Panksepp, 1998). He described the SEEKING system as the “granddaddy” of all his emotional systems in that it is able to regulate those systems (Kirsch, 2019). In an effort to describe the neurobiological underpinnings of Panksepp's idea that the SEEKING system regulates the six emotional systems, Kirsch looked to indirect mechanisms by which the lateral hypothalamus affects brain areas associated with RAGE, FEAR, PANIC, etc. Orexinergic neurons of the lateral hypothalamus modulate appetite and arousal (which relate to the Freudian motivational drives hunger and sleep). Kirsch described that orexinergic neurons have receptors for ghrelin and estrogen, which have an excitatory effect on said neurons, as well as adenosine and angiotensin II, which have an inhibitory effect on said neurons (2019). Interestingly, testosterone receptors were not found to be present on the orexinergic neurons of the lateral hypothalamus, but given that testosterone is enzymatically converted to the estrogen derivative estradiol, a hormone that also influences male sexual behavior, estrogen can be thought of as a substitute for testosterone as the imperative motor factor influencing sex drive (Kirsch, 2019). These orexinergic neurons were found to project out to all of the brain areas associated with Panksepp's emotional systems [i.e., the ventral tegmental area (VTA) and nucleus accumbens, which again are associated with the SEEKING system, the medial amygdala that is associated with RAGE, the central amygdala that is associated with FEAR, and so on] (Kirsch, 2019). This supports Panksepp's assertion that the SEEKING system is the “granddaddy” of the other emotional systems. It makes logistical sense for there to be a system that modulates the importance of particular emotions and instinctual needs, as it is important to be able to prioritize instincts over emotions, as well as one drive over another (Kirsch, 2019). For example, if one wants to sleep, it is important to be able to turn off RAGE, and if one is both hungry and thirsty, it is important to prioritize thirst, given that one can survive 21 days without food and only 3 days without water (Kirsch, 2019).

The six instinctual systems then do not depend on hormones lodging in the lateral hypothalamus. They are turned on directly by orexigenic neurons. We now have three levels of motivational factors:

- Dopaminergic SEEKING; as Panksepp termed it, “The goad without a goal.”
- Hormonally sponsored drives that orient SEEKING to a specific goal that includes cathected humans.
- Orexigenic instincts that have either a pleasant feel, CARE, LUST, and PLAY, or an aversive quality, RAGE, FEAR, and PANIC.

### The Opioid Hormonal System

The endogenous opioid system is best conceived as a hormonal system as endogenous opioids produced by the brain and adrenal cortex circulate widely in the body, have a multitude of receptor sites, and influence a variety of bodily processes (Johnson et al., 2014). Opioid receptors are prevalent in the gut, as evidenced by the constipation produced by exogenous opioid administration and the cramping, diarrhea, and vomiting characteristic of opioid withdrawal. Endogenous opioids play a role in activating the immune system, whereas exogenous opioid administration has been demonstrated to suppress immune function (Plein and Rittner, 2018). Endogenous opioids interact with other hormonal systems. The administration of exogenous opioids disrupts function in the hypothalamic–pituitary–adrenal axis and the hypothalamic–pituitary–gonadal axis resulting in decreased production of sex hormones (Khademi et al., 2015). Opioids are also responsible for modifying the intensity of pain.

The regulation of pain by the opioid system has been adapted to modulate social interaction and relatedness (Stein et al., 2007; Panksepp and Watt, 2011). The opioid hormonal system is the engine of relatedness. The hedonic feelings generated by endogenous opioid stimulation serve to motivate humans to continue to engage in social behavior (Panksepp, 1998). Human connection maintains opioid tone in a pleasurable window; too little contact hurts as does too much. Low opioid tone compels us to seek out human contact. Conversely, high opioid tone prompts a desire for distance from others. This is why it feels so nice to interact with a partner or friends after spending some time alone and why after a busy day around colleagues at work or an evening at a crowded party, quietly reading or watching television alone feels blissful (Johnson et al., 2014). We use engineering models to treat our patients and offer an updated version of our inverse U function of pain/pleasure and endogenous opioid tone here:

### The Effect of Addictive Drugs on SEEKING, Drive, Instinct, Relatedness, Pleasure, and Unpleasure

**Figure 1:**
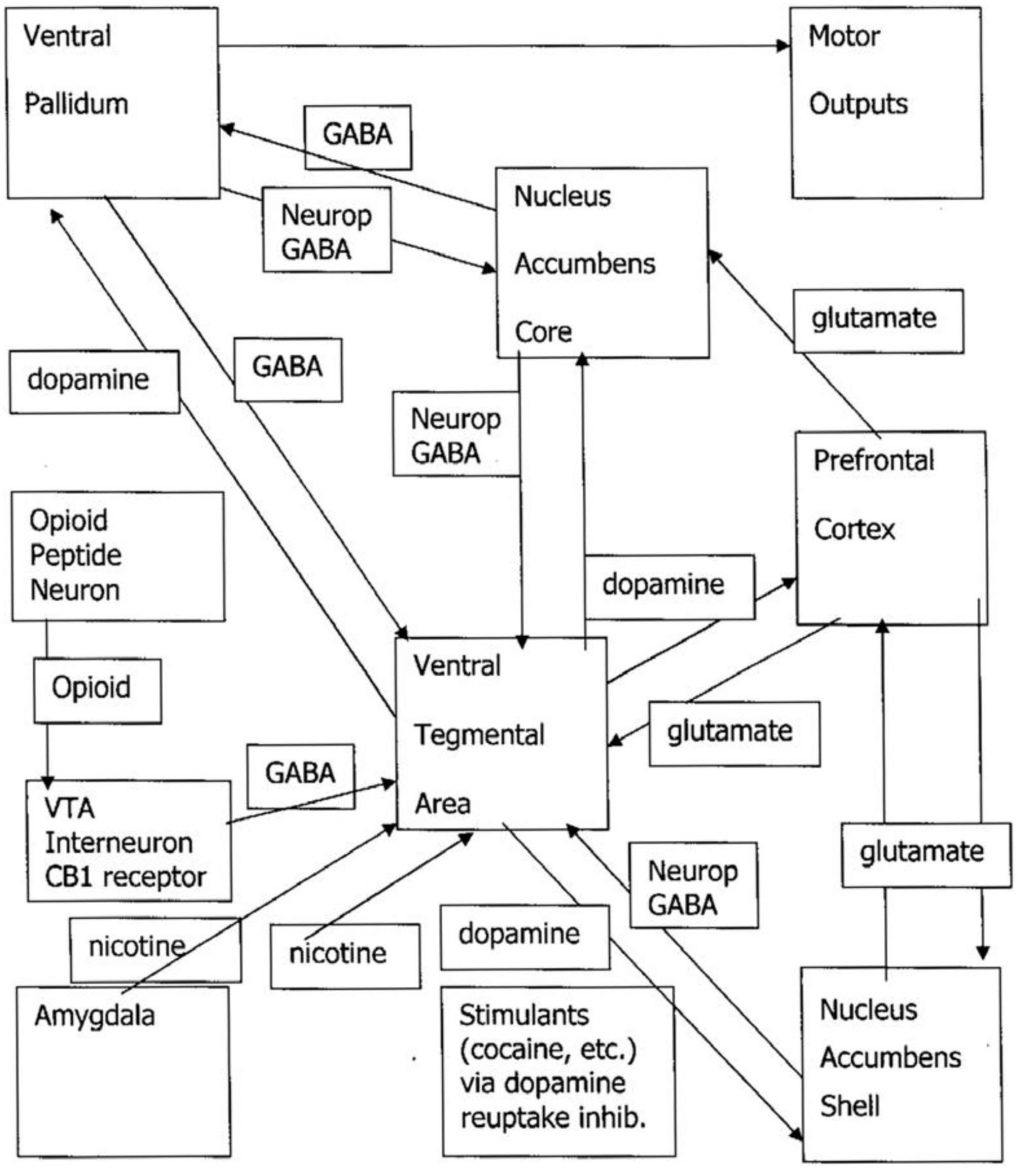
Drug craving/drug dreaming pathways. VTA, ventral tegmental area; GABA, gamma-aminobutyric acid; neurop, neuropeptide; NAS, nucleus accumbens shell. The upper drug pathway is the triangle of the VTA/NAS/prefrontal cortex. The downer drug pathway is represented by the opioid peptide neuron removing the tonic GABAergic brake on VTA release of dopamine to the NAS.

We begin with a previously published engineering model of the SEEKING system as influenced by addictive drugs (Johnson and Faraone, 2013; Johnson et al., 2014; Johnson, 2018). Nicotine, amphetamine, and cocaine are the “upper” group. They directly trigger dopamine barrages from the VTA to the nucleus accumbens shell (NAS). Opioid and cannabis directly inhibit the GABAergic inhibitory neuronal system that is a tonic brake on dopamine release to the NAS. Alcohol and benzodiazepines are GABAergic, so ingestion shuts off the appetite for more. For example, one may notice that after a drink or two, one feels one has had enough. Persons who use alcohol for “relief” from PANIC, FEAR, and RAGE signals may override the natural inhibition of “enough” to get rid of the dysphoric feelings, cause the downregulation of the gamma-aminobutyric acid (GABA) system and the upregulation of balancing glutamatergic and noradrenergic drivers, and induce withdrawal when the drug is stopped. The flood of uninhibited dopamine is then responsible for opponent process of the downregulation of the dopamine system combined with new onset of drinking dreams, SEEKING the drug while motor inhibition is activated during rapid eye movement (REM) sleep (Johnson, 2001). N.B.—this formulation is based on Figure 1. The authors appreciate that opinions and evidence on alcohol and dopamine are mixed. See, for example, (Thiruchsevam et al., 2017).

The SEEKING system is directly taken over by addictive drugs. An earlier paper explained that Freud's concept of “the will” was simply SEEKING operating inside us (Johnson, 2013). Once SEEKING for addictive drugs is in control of the will, instinctual systems, such as CARE, LUST, PLAY, FEAR, PANIC, and RAGE, turn on sluggishly. While it is impossible to completely avoid sleep, it turns on sluggishly because of downregulated dopamine function, resulting in insomnia.

These instinctual systems underlie relatedness. If one is trying to relate to an individual who SEEKS one or more drugs, one becomes aware of their need to disengage to inhale a cigarette, have a drink, or that the individual returns home only after all the money for cocaine has been spent.

Addictive drugs also cause a surge of serotonin:
Once the SEEKING system is made hyporesponsive to Freudian drives, such as food, water, sex, sleep, and relationships, due to downregulated dopaminergic tone, they are sluggishly active while dopamine-releasing drugs are sought because they will move tone back toward normal. There is an additional explanation from the perspective of central 5-hydroxytryptamine (serotonin) turnover. With the exception of benzodiazepines, all drugs with addictive potential have the capability to induce the release of 5-hydroxytryptamine in the nucleus accumbens (Table 2), i.e., a core region of the SEEKING system. An increased level of 5-hydroxytryptamine decreased Freudian drives by decreasing the dopaminergic tone and attachment urges by releasing beta-endorphin (Kirsch and Buchholz, 2020). Hence, drug-addicted individuals complain of insomnia, they lose weight, and they favor drugs over relationships, even with partners and children.

**Table 2:** Release of serotonin caused by drug use.

**Addictive drug**	**Reported 5-HT release in the nucleus accumbens**
Cocaine	Broderick et al., 1993; Parsons and Justice, 1993; Teneud et al., 1996
Alcohol	Yoshimoto et al., 1992
Nicotine	Ma et al., 2005
Amphetamine	Hernandez et al., 1987; Kankaanpää et al., 1998
MDMA	Kankaanpää et al., 1998; Trigo et al., 2007; Baumann et al., 2008
Morphine and heroin	Tao and Auerbach, 1995; Solms and Turnbull, 2002/18; Fadda et al., 2005; Stein et al., 2007; Watt and Panksepp, 2009; Thiruchsevam et al., 2017; Tabi et al., 2020

**Figure 2:**
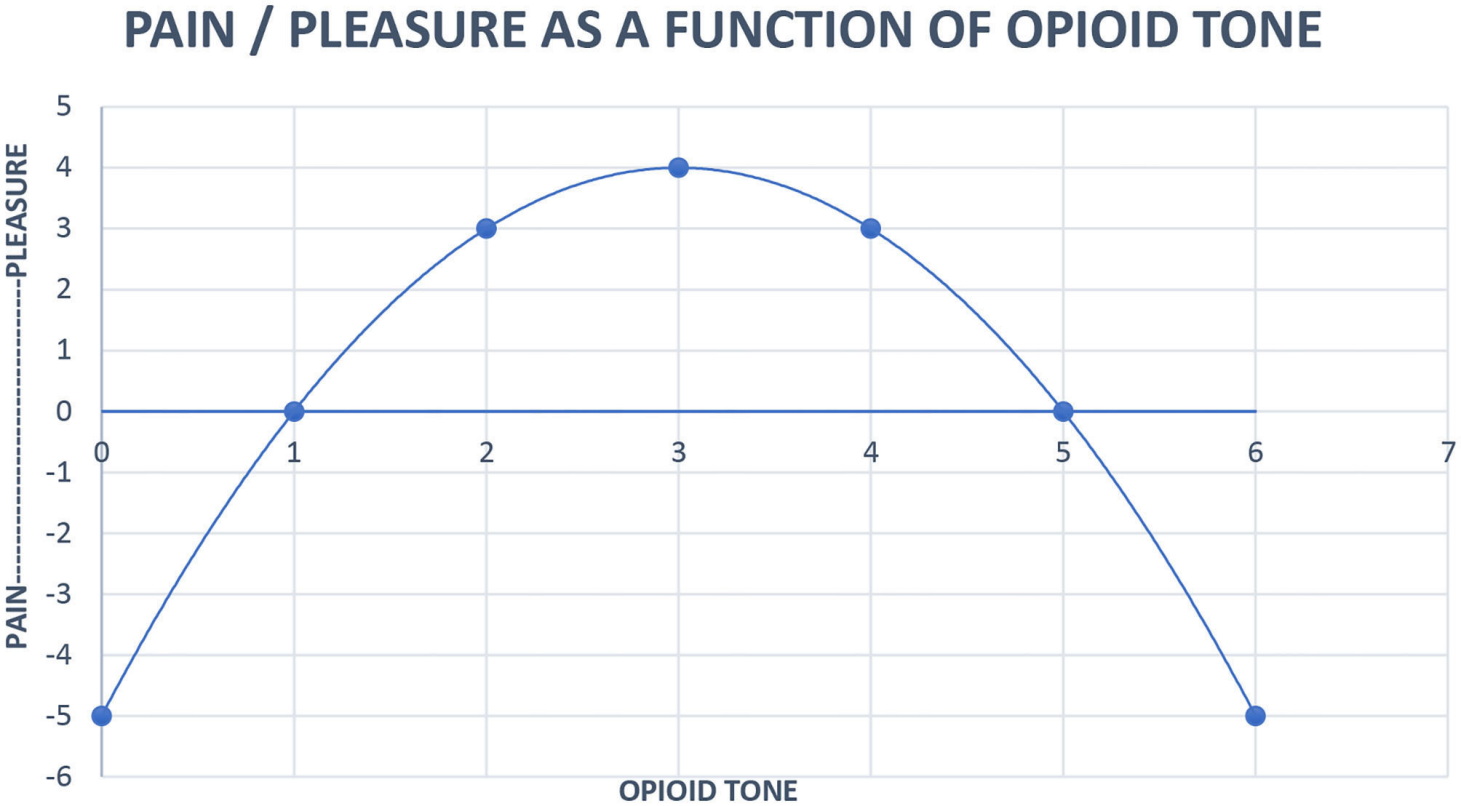
Neurobiological systems engineering model of the relationship of pain, pleasure, and opioid tone. Pleasure (x) = 4 – (x – 3)^2^. *x* = opioid tone, limit *x* = 0 < x < 6.

Work on our neuropsychoanalytic addiction service provides further evidence for the opioid hormonal system being the driver of relatedness and for human contact being the treatment for addiction. As shown in Figure 2, buprenorphine maintenance moves patients to the right side of the inverse U where emotional contact hurts. Patients maintained on opioids for addiction relate to others autistically. They often exhibit gaze avoidance, report that they have nothing to talk about, and their strongest wish is often to be seen as infrequently as possible. These experiences reflect evidence by Carroll and Weiss (2017) that psychotherapy with patients on buprenorphine maintenance is ineffective; their opioid tone is too high, their drive to relate is squashed, and human contact hurts too much. In fact, we have been able to craft a neuropsychoanalytically informed version of contingency management for buprenorphine-maintained pregnant women where the reward for being off other addictive drugs is to disengage from psychotherapy. This approach both eliminates the need to treat neonatal abstinence syndrome (Tabi et al., 2020) and confirms that human contact hurts buprenorphine-maintained pregnant women.

Patients on opioid maintenance are not aware that they are unrelated. It is only evident to others around them. Patients who are helped off opioids suddenly feel the rush of anxiety associated with low opioid tone. Suddenly feeling alone and vulnerable to emotions that they did not have on opioids acts as a driver toward relapse. They long for the feeling of connectedness that they had on opioids. We call it, “A person in a pill.” To counteract the feeling of vulnerability, patients coming off opioids are seen every day on our addiction medicine service while going through withdrawal. Frequent human contact works against relapse by satisfying the drive for relatedness (Johnson et al., 2014).

The current standard of care according to the American Society for Addiction Medicine (ASAM) for treating opioid use disorder (OUD) is medication-assisted treatment (MAT), yet little is known about the potential effects of MAT on drive, instinct, and relatedness. Both buprenorphine and methadone are unquestionably lifesaving medications as evidenced by their effect on reducing mortality by 50% (Coffa and Snyder, 2019). Unrelatedness may explain in part why the current buprenorphine prescribing guidelines were updated to remove the requirement that all patients seeking MAT for OUD must participate in mandatory individual and/or group therapy. Making this a requirement causes dropouts from maintenance programs, as counseling was found to have only marginal benefits for buprenorphine-maintained patients, and when combined with the inadequate access to behavioral health providers, access to MAT became drastically limited (Martin et al., 2018). It may be that simply dropping the requirement for psychotherapy recognizes reality without having an explanation for why this is needed.

Our understanding from our patients about what they mean by “high” is simply normal functioning (Johnson, 2018). In other words, “high”-functioning people constantly have the pleasure of bonding and human contact in a propitious human environment. Persons with PANIC, RAGE, and/or FEAR turned on use drugs to get rid of the signal that they need to modify their human environment to feel better.

When meeting a new person, the question is, “What kind of person is this? Should I form a bond?” The answer is not a routine exercise of current judgment. Rather, it is based on previous cathexes. Persons with early histories of benevolent relationships are prone to making more propitious relationships, and persons with a history of abuse are prone to making new relationships based on their early abuse histories (Johnson, 2008).

### Brief Neuropsychoanalytic Case

Mr. X is a 34-year-old man with post-traumatic stress disorder, narcissistic personality disorder, alcohol, opioid, cocaine, amphetamine use disorder, alcohol-induced chronic pancreatitis, and opioid-induced hyperalgesia. His cold pressor time, the time he can keep his forearm immersed in a painful ice water bath, has ranged from 14 s when he is using street opioids to 3 min after a 3-month course of low-dose naltrexone to fix the opioid system damage from exogenous hormone (Oaks et al., 2018). Additional psychosocial factors contributing to his overall illness severity include complicated bereavement due to the murder of his wife and young daughter by drug dealers, unemployment despite high educational obtainment, a history of severe physical and emotional abuse during childhood, and ongoing emotional abuse by his immediate family members.

Mr. X has been a patient on our neuropsychoanalytic service for 6 years. His treatment course has included twice weekly individual transference-based psychotherapy and disulfiram for severe alcohol cravings given his high risk of mortality from resumption of alcohol use in the setting of recurrent alcoholic pancreatitis. Later in his course of treatment, at the request of his mother following a period of relapse on heroin and cocaine, Mr. X agreed to 20 mg/day buprenorphine maintenance for a year. During this time, he was objectively disengaged during his individual sessions and actively avoided discussion of anything more than superficial topics. He continued during this time to have significantly impaired interpersonal and occupational functioning. He reported numerous occasions where he had voluntarily left home to sleep unsheltered on the street specifically to avoid interacting with his mother and other family members.

Mr. X reported feeling “mentally sluggish and emotionally numb.” He asked to discontinue buprenorphine maintenance. A major concern about remaining on MAT specifically cited was that he was, “Unable to feel a connection to or feel love from my fiancé.”

With detox from buprenorphine and low-dose naltrexone, he demonstrated a significantly different behavior in session. He subjectively reported improvements in his cognition, restoration of his ability to experience emotions (both positive and negative), could experience his fiancé's love and affection again, and had returned home to his mother's house. Mr. X demonstrated better engagement in the therapeutic discussion, openly shared his thoughts and feelings related to the pain of his prior life experiences, and was receptive to the treating provider's interpretations of his defense mechanisms and confrontation of cognitive distortions. His affect became brighter and less constricted, and he became employed again. Mr. X did continue to have some interpersonal difficulties at work but was able to tolerate social interactions with his coworkers. He demonstrated a restoration in his relatedness following cessation of his buprenorphine maintenance and continues in treatment. Several months after his detox, Mr. X briefly relapsed on alcohol and street fentanyl following a narcissistic injury, but when offered to re-start MAT, he adamantly refused stating, “On buprenorphine I couldn't feel anything, I didn't care about anything, and I felt like I was losing my intellect. I need to be able to tolerate my feelings without needing drugs. I want to feel love from my fiancé.”

## Discussion

There has been a long running discussion in psychoanalysis about drive and instinct. Are they the same? Did translation blur some distinction Freud had in mind? Did he use one word at times, and the other at other times, as if he thought them interchangeable? Is the concept of drive outdated for current psychoanalysis? Should we take a relational stance that what we see between psychoanalyst and patient is the bedrock of our science? We have revisited this question with a neuropsychoanalytic approach. What we observe clinically should line up with neuroscience.

Our neuropsychoanalytic addiction medicine service gives us additional information. The brains of our patients have been changed by addictive drugs in ways that are characterized by neuroscience investigations, resulting in aberrant behaviors that we constantly ask patients to describe.

This has resulted in a revision of motivational factors where neuroscience and clinical observation line up in a new way. In summary:

- Dopaminergic SEEKING is a tonic stimulus to exploration; as Panksepp termed it, “The goad without a goal.”
- Hormonally sponsored drives orient SEEKING to a specific goal that includes cathected humans.
- Orexigenic instincts have either a pleasant feel, CARE, LUST, and PLAY, or an aversive quality, RAGE, FEAR, and PANIC.
- Addictive drugs take over SEEKING, orienting the victim to constantly, when prompted by withdrawal, endeavoring to obtain a dopamine surge from drug use.
- The “relief” of drug use, the “high,” is having the drug remove the aversive experience of PANIC, RAGE, and/or FEAR.

Freud's original conflation about pleasure and unpleasure (Johnson, 2017) is separated into:

- Pleasure is a relaxed, slow, mostly opioidergic experience that has no pressure to repeat. It is modulated interpersonally to optimize human contact.
- Unpleasure is the experience of drives, including for drugs if addicted, not being gratified. One is tortured by the unmet wish for sex, sleep, food, water, or drugs.

High functioning people have all their basic drive needs met. This may be most evident when one is on vacation with a partner and some friends, free to spend time socially, or read a book alone when one wants the pleasure, as indicated in Figure 2, of optimal human contact.

SEEKING is fundamentally dopaminergic. It is turned on all the time, even when one is asleep and dreaming. SEEKING is subserved by other systems. Without a switching system, SEEKING is not tuned to any particular goal.

By virtue of the anatomy with the VTA to the NAS median forebrain bundle running through the lateral hypothalamus, drives are turned on by hormones and turned off by a surge of serotonin. Cathexis requires sequential engagement of dopamine and then oxytocin and endogenous opioid. One does not respond to strangers who brush by you on the street. Cathexis requires emotional engagement. Orgasm with a new lover causes a surge of oxytocin and endogenous opioid that promotes cathexis (Johnson, 2008). One may remember lovers much more than acquaintances.

PANIC, RAGE, and FEAR are uncomfortable instincts that promote survival. For teenagers who have had them turned on for their entire lives, the discovery of addictive drugs that turn off these instinctual systems tempts them to be their own psychiatrist and “self-medicate” those instincts away.

The fact that SEEKING has been corrupted by drugs must be made unconscious to allow continued drug use despite the danger that is apparent to treaters. Defenses, such as splitting, omnipotent control, denial, minimization, avoidance, and rationalization, neutralize messages that drug use is dangerous. Rather than say, “My brain has been taken over by sellers of tobacco,” users say, “I like smoking cigarettes. It relaxes me!” The need for a cigarette is a product of dopaminergic craving fueled by nicotine withdrawal. It is unpleasure relieved by another episode of drug use. Interpretations of defenses may undermine the denial system and help actively addicted patients decide to stop using addictive drugs.

Addictive drugs cause dopamine barrages to the nucleus accumbens accompanied by surges of serotonin. This makes it difficult or impossible, depending on which drug it is, for actively addicted patients to make a cathexis with treaters, the engine of psychotherapy. It allows us to understand that for some drugs, the transference relationship can only be used for treatment after use has stopped and the SEEKING system is now available for the patient to relate to the treater.

Pleasure is a relaxed, slow moving hormonal response to events, especially proximity of cathected friends. One wants to be around friends until it feels like enough, signaled unconsciously by opioid tone moving past the midpoint of Figure 2. One then wants to be alone until opioid tone moves back past the midpoint where proximity is once again going to produce pleasure. The dysphoria of low opioid tone may be the source of the aphorism of AA, “When you don't feel like a meeting, you should go to a meeting.” The proximity of AA members promotes the relaxed good feeling of optimal opioid tone.

With this substantial discussion about how SEEKING, drive, instinct, pleasure, and unpleasure work, and how drugs affect these neurobiological systems, we are now in a position to answer our initial questions about mass killing and the place of psychoanalysis in addiction treatment. PANIC, FEAR, and RAGE are uncomfortable for all of us. Teenagers who grow up in environments where these instinctual systems are turned on all the time use drugs to turn off the signals of distress. While one might hope that children who mature in distressing environments look for better relationships to help them, their cathexis system is tuned to abuse. Drugs and bad relationships go together. Many addicted patients have difficulties with relatedness that may be ameliorated by a treatment where relatedness is the central issue that is studied with the treater. This is in marked contrast with addiction treatments that involve “counseling” or “skills training.” We regard our treatment as a sophisticated version of “12 step facilitation.” Our psychoanalytic style includes transference focused psychotherapy confrontations, such as, “Most people with good recovery go to Alcoholics Anonymous and you are not going. What is your thinking?”

In order to accommodate the mass killing involved, outside observers also employ defenses. For example, tobacco consumption is described by “packs smoked” rather than “cigarettes inhaled.” Smoking “a half pack per day” is a description used by traumatized physicians who experience constant deaths from tobacco. The defense is minimization. The more powerful, disconcerting and danger-announcing alternative would be, “Inhaling 10 cigarettes per day.”

Allotting responsibility to the individual “abusing” a drug shifts responsibility for mass killing from the addictive drug industry to their victims. In an unfortunate consilience, since the drug takes over the will, the victims of mind-controlling drugs also take responsibility for using a drug that, in most cases, took over their SEEKING system during childhood. Psychoanalysis can also be used to understand the social phenomenon of tolerating mass killing in that most individuals also use defenses to make the deaths constantly around them the product of the weak will of the victims rather than the malevolence of profit-driven drug selling. Use of these defenses against conscious awareness immobilizes the need for life-saving responses.

On our neuropsychoanalytic addiction medicine service, we have much supervision to address the helplessness we feel while some of our patients continue to use potentially lethal drugs. Our awareness also promotes public health and political activism. We suggest that using psychoanalysis for drug treatment is responsible for more distress in treaters and better outcomes for patients. We appreciate that this assertion is a product of a perspective rather than any empirical evidence.

This exposition is an example of the use of psychoanalysis as a general psychology. It is useful for treatment. We have also used psychoanalysis to make models that explain addictive disease, addictive behavior, and public health issues.

## Data Availability Statement

The original contributions presented in the study are included in the article/supplementary materials, further inquiries can be directed to the corresponding author/s.

## Ethics Statement

Written informed consent was obtained from the individual(s) for the publication of any potentially identifiable images or data included in this article.

## Author Contributions

BJ: wrote a shell/outline of the manuscript. TR, LC, BF, and MK: wrote sections of the manuscript that were then synthesized by BJ. All authors contributed to the article and approved the submitted version.

## Conflict of Interest

The authors declare that the research was conducted in the absence of any commercial or financial relationships that could be construed as a potential conflict of interest.

## Footnotes

^1^https://www.cdc.gov/nchs/fastats/deaths.htm.

^2^https://www.cdc.gov/tobacco/data_statistics/fact_sheets/fast_facts/index.htm#:~:text=Cigarette%20smoking%20is%20responsible%20for,or%201%2C300%20deaths%20every%20day.&text=On%20average%2C%20smokers%20die%2010%20years%20earlier%20than%20nonsmokers.

^3^https://www.cdc.gov/alcohol/features/excessive-alcohol-deaths.html.

^4^https://www.cdc.gov/drugoverdose/data/statedeaths.html#:~:text=In%202018%2C%2067%2C367%20drug%20overdose,driver%20of%20drug%20overdose%20deaths.
